# Achieving Synergy: Linking an Internet-Based Inflammatory Bowel Disease Cohort to a Community-Based Inception Cohort and Multicentered Cohort in Inflammatory Bowel Disease

**DOI:** 10.2196/jmir.5655

**Published:** 2016-06-03

**Authors:** Bharati Kochar, Molly Aldridge, Suzanne Follan Cook, Renee Bright, Meaghan Mallette, Heather Moniz, Samir A Shah, Neal S LeLeiko, Jason Shapiro, Bruce E Sands, Wenli Chen, Elizabeth Jaeger, Joseph Galanko, Millie D Long, Christopher F Martin, Robert S Sandler, Michael D Kappelman

**Affiliations:** ^1^University of North CarolinaCenter for Gastrointestinal Biology and DiseaseChapel Hill, NCUnited States; ^2^GlaxoSmithKlineResearch Triangle Park, NCUnited States; ^3^Rhode Island HospitalProvidence, RIUnited States; ^4^The Warren Alpert Medical School at Brown UniversityProvidence, RIUnited States; ^5^Hasbro Childrens' HospitalDivision of Pediatric Gastroenterology, Nutrition and Liver DiseasesProvidence, RIUnited States; ^6^Mt. Sinai Medical CenterDivision of GastroenterologyNew York City, NYUnited States

**Keywords:** Crohn’s disease, ulcerative colitis, inflammatory bowel disease, cohort study

## Abstract

**Background:**

Traditional cohort studies are important contributors to our understanding of inflammatory bowel diseases, but they are labor intensive and often do not focus on patient-reported outcomes. Internet-based studies provide new opportunities to study patient-reported outcomes and can be efficiently implemented and scaled. If a traditional cohort study was linked to an Internet-based study, both studies could benefit from added synergy. Existing cohort studies provide an opportunity to develop and test processes for cohort linkage. The Crohn’s and Colitis Foundation of America’s (CCFA) Partners study is an Internet-based cohort of more than 14,000 participants. The Ocean State Crohn’s and Colitis Area Registry (OSCCAR) is an inception cohort. The Sinai-Helmsley Alliance for Research Excellence (SHARE) is a multicentered cohort of inflammatory bowel disease patients. Both the later cohorts include medical record abstraction, patient surveys, and biospecimen collection.

**Objective:**

Given the complementary nature of these existing cohorts, we sought to corecruit and link data.

**Methods:**

Eligible OSCCAR and SHARE participants were invited to join the CCFA Partners study and provide consent for data sharing between the 2 cohorts. After informed consent, participants were directed to the CCFA Partners website to complete enrollment and a baseline Web-based survey. Participants were linked across the 2 cohorts by the matching of an email address. We compared demographic and clinical characteristics between OSCCAR and SHARE participants who did and did not enroll in CCFA Partners and the data linkage.

**Results:**

Of 408 participants in the OSCCAR cohort, 320 were eligible for participation in the CCFA Partners cohort. Of these participants, 243 consented to participation; however, only 44 enrolled in CCFA Partners and completed the linkage. OSCCAR participants who enrolled in CCFA Partners were better educated (17% with doctoral degrees) than those who did not (3% with doctoral degrees, *P*=.01). In the SHARE cohort, 436 participants enrolled and linked to the Partners cohort. More women (60% vs 50%) linked and those who linked were predominantly white (96%; *P*<.01). Crohn’s disease patients who linked had lower mean scores on the Harvey-Bradshaw Index (3.6 vs 4.4, *P*<.01). Ulcerative colitis patients who linked had less extensive disease than those who did not link (45% vs 60%, *P*<.01).

**Conclusions:**

Linkage of CCFA Partners with cohorts such as OSCCAR and SHARE may be a cost-effective way to expand the infrastructure for clinical outcomes and translational research. Although linkage is feasible from a technical, legal, and regulatory perspective, participant willingness appears to be a limiting factor. Overcoming this barrier will be needed to generate meaningful sample sizes to conduct studies of biomarkers, natural history, and clinical effectiveness using linked data.

## Introduction

Cohort studies are designed to evaluate risk factors for developing a health condition and/or factors which affect the natural history of disease [1]. In traditional cohort studies, subjects are extensively characterized at baseline (eg health history, environmental exposures), often with the collection of biosamples and observed over time for the development of outcomes of interest. These outcomes usually include important medical events such as surgery, cancer, or death. In recent years, there has been growing interest in the use of the Internet and social media to develop electronic cohorts, which focus on patient-reported exposures and health behaviors and patient-reported outcomes (PROs) such as fatigue, well-being, and illness experience [2-5]. Electronic cohorts have the advantage of efficient recruitment and data collection and may be less costly to maintain over time. Linking a traditional cohort with an electronic cohort offers an opportunity to derive the benefits of both cohort designs. This hybrid cohort would have the benefit of well-characterized, validated cases and biological specimens. The speed and low cost of the electronic component would provide a cost-effective approach to evaluate an extended array of PROs. Translational studies could exploit the biological samples in the hybrid cohort.

Two novel and complementary cohort studies initiated by the Crohn’s and Colitis Foundation of America (CCFA) provide an opportunity to develop and test the process of cohort linkage. The Ocean State Crohn’s and Colitis Area Registry (OSCCAR), a traditional cohort study, is an inception cohort that includes medical record abstraction, patient surveys, and biospecimen collection. CCFA Partners is an electronic cohort of more than 14,000 participants with inflammatory bowel diseases (IBDs) that focuses on patient-reported exposures and outcomes. In addition, IBD patients enrolled in the Sinai-Helmsley Alliance for Research Excellence (SHARE), a 7-center prospective observation cohort, were also evaluated to test the linkage of a traditional cohort to an electronic cohort. As these cohorts have different areas of focus, they are complementary to one another. If successful, linkage would provide a cost-effective strategy to expand the data assets of both cohorts, enabling studies not possible in either cohort alone. Record linkages of this kind have the potential to create information-rich environments [6] allowing for a more patient-centered and holistic approach to observational research, patient care, and improved patient outcomes.

Other record linkage initiatives have been established around the world for a variety of research purposes. For example, Australia’s Population Health Research Network is expected to be the world’s “largest population database supporting health research, policy, and planning” and links together a number of health administrative databases [7]. Researchers in Canada are working to link health administration data with data from the Canadian Longitudinal Study on Aging cohort and the Canadian Partnership for Tomorrow Project cohort [8]. An initiative in the United Kingdom aimed to link PRO measures reported through an Web-based data collection system to clinical data in a cancer registry [9]. To our knowledge, our study represents the first published attempt to link data from a traditional, inception cohort to a large electronic cohort.

The objectives of this project were (1) to develop the technical, regulatory, and legal infrastructure to corecruit OSCCAR and SHARE participants and into CCFA Partners and link the data assets of these 2 cohorts and (2) to evaluate the feasibility of this corecruitment and linkage process. If successful, linkage of these cohorts would produce a “virtual database” of expanded clinical, PROs, and biospecimen data.

## Methods

### Study Setting

#### OSCCAR

OSCCAR is a novel, community-based inception cohort of 408 Crohn’s disease (CD) and ulcerative colitis (UC; IBD subtypes) patients launched in Rhode Island in 2008 and closed to enrollment in 2013. Funded by the Centers for Disease Control and Prevention, investigators at Mount Sinai Hospital, Harvard Medical School, and the Warren Alpert Medical School of Brown University jointly manage the OSCCAR cohort. The cohort is designed to collect data on incidence rates of CD and UC, disease outcomes, and factors predictive of disease onset and outcomes. In addition to the prospective and longitudinal collection of clinical data by physician report and chart abstraction, OSCCAR participants complete regular surveys and contribute to a biobank of blood, urine, and stool specimens [10].

Newly diagnosed pediatric and adult patients with IBD who were residents of Rhode Island were referred by their diagnosing gastroenterologist or surgeon for enrollment in OSCCAR from January 2008 to January 2013. After informed consent, diagnosis of IBD was confirmed by chart review by medically trained study personnel according to standard criteria of the National Institutes of Diabetes and Digestive and Kidney Diseases IBD Genetic Consortium. Participants were visited at study enrollment and requested to provide blood, urine, and stool samples. All subjects were also asked to complete the Inflammatory Bowel Disease Questionnaire, IMPACT Questionnaire, Short Form Health Survey (SF)-36, EuroQoL 5, Work Productivity and Activity Impairment Questionnaire, Functional Assessment of Chronic Illness Therapy, Short Inflammatory Bowel Disease Questionnaire, An Assessment of Environmental Risks for Ulcerative Colitis and Crohn’s Disease in the United States, and PHQ-8 Depression Diagnostic and Severity Measure [11-17]. Subjects with CD were asked to complete the Harvey-Bradshaw Index (HBI). Subjects with UC were asked to complete the Simple Clinical Colitis Activity Index (SCCAI). The participants are contacted every 6 months through the study period to update their profiles and collect specimens [10, 18].

#### Share

The SHARE is a prospective observational cohort of patients with IBDs. The cohort was established with the goal of creating a database of clinical information and a repository of biological specimens for genetic, molecular, and microbiological research to better understand IBD and to help develop new therapies. Starting in July 2012, patients were recruited from 7 academic centers around the United States: Mount Sinai Hospital, University of Chicago Medical Center, Massachusetts General Hospital, Cedars-Sinai Medical Center, Mayo Clinic, Rochester, University of North Carolina at Chapel Hill Hospital, and Washington University, Saint Louis, Medical Center. Enrollment is currently ongoing.

Patients who are aged younger than 18 years, unable to understand or provide informed consent, and do not have a confirmed diagnosis of IBD in their medical records were excluded. Recruited patients provided demographic information, medical history, surgical history, family history, medication use, extraintestinal manifestations, and blood samples via an interview with a study coordinator. Disease characteristics, medication use, extraintestinal manifestations, and laboratory results are obtained from the medical record by the study coordinator. The HBI, SCCAI, and Modified Pouchitis Disease Activity Index are completed during the study visit by the investigator or a study coordinator [19-21]. The Depression Activity and Severity Index (PHQ-8) and Manitoba Index are completed by the patient during the study visit [17, 22].

#### CCFA Partners

CCFA Partners is a validated, Internet-based, long-term cohort study of adult subjects with IBD initiated in July 2011 and administered by the University of North Carolina, Chapel Hill. The development of CCFA Partners has been described in detail previously [23]. In brief, participants with a self-reported diagnosis of UC, CD, or indeterminate colitis were recruited through CCFA email rosters, the CCFA website, social media outlets, and at educational and fundraising events. The baseline and subsequent surveys administered every 6 months include questions regarding demographics, disease type, disease course and activity, IBD-related treatments, and concurrent medication use including alternative medicines, family history of disease, diet, exercise, and PROs including quality of life and medication adherence [23]. To date, over 14,000 people with IBD have been enrolled. Published and planned studies using data generated from the CCFA Partners e-cohort include the evaluation of (1) the impact of IBD on important, patient-centered outcomes, (2) the role of environmental influences (eg, sleep, depression, diet, exercise) on disease activity, (3) patient perceptions on selected topics such as quality of care, biobanking, and so forth, and (4) clinical effectiveness and drug safety research [24-29]. Further details on the validation of patient-reported data in a subset of CCFA Partners are published [30].

### Linkage Process

#### Eligibility

To enable record linkage, we identified members of OSCCAR and SHARE who met the criteria for membership in CCFA Partners, including having a diagnosis of IBD and access to the Internet.

#### Education and Recruitment

OSCCAR participants are seen in person at years 1 and 5 after enrollment and are contacted by phone every 6 months. During these points of contact, OSCCAR staff provided participants with recruitment materials about the CCFA Partners cohort and the OSCCAR-CCFA Partners linkage study. Participants were also provided with the CCFA Partners Web address for additional information. All OSCCAR participants were contacted on at least three occasions.

SHARE participants were asked if they were interested in participating in CCFA Partners at the time of their enrollment in SHARE between July 2012 and December 2014. Cedars-Sinai Medical Center did not participate in this linkage study.

#### Linkage

Participants were linked across the 2 cohorts by the matching of their email addresses. After informed consent for the data linkage, participants were sent an automated email with instructions on how to access the CCFA Partners website to complete CCFA Partners enrollment/consent and to fill out the baseline survey. Each participant was sent up to three email reminders. In addition, in the OSCCAR cohort, the study coordinator and research assistants sent personalized letters and made phone calls to each individual to encourage completion of the linkage process.

#### Ethics

The study protocol was approved by the Institutional Review Boards at Rhode Island Hospital, the University of North Carolina at Chapel Hill, and the Institutional Review Boards of the SHARE sites that participated. Personal health information was protected across linkages by using deidentified study identification numbers. Where necessary, personal identifiers for consented participants were made available to trained research staff identifiers through a password-protected Web portal.

#### Analysis

The enrolled subjects were compared to the unenrolled subjects. Categorical variables were compared using Fisher’s exact tests or chi-square tests as appropriate and continuous variables were compared using t tests. All P values less than .05 were considered statistically significant. Analyses were performed using SAS 9.4 (SAS Institute, Cary, NC) and Stata 14.0 (StataCorp, College Station, TX).

## Results

### OSCCAR

**Figure 1:**
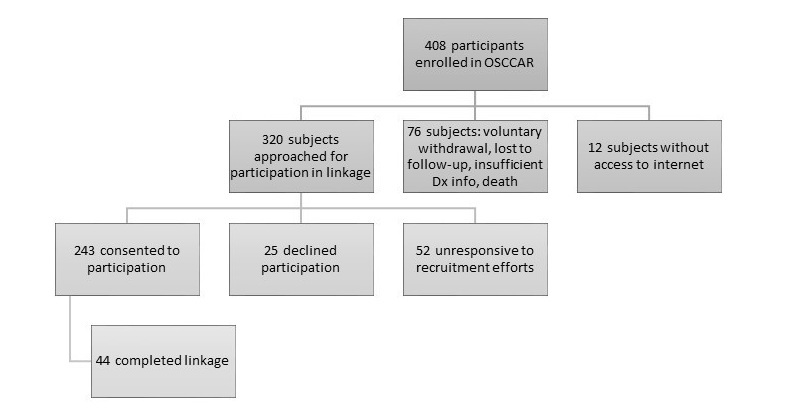
Recruitment efforts for the CCFA Partners/OSCCAR record linkage.

Figure 1 illustrates the recruitment process for the CCFA Partners/OSCCAR linkage. A total of 408 participants have been enrolled in OSCCAR. Seventy-six of these subjects are no longer being followed because of voluntary withdrawal, loss to follow-up, criteria insufficient for a diagnosis of IBD, or death. An additional 12 subjects report not having access to the Internet. Of the remaining 320 participants who were eligible for linkage, all were approached for participation. Two hundred forty-three subjects consented to linkage with the Partners cohort, 25 declined participation, and the remaining 52 did not respond to recruitment efforts. Of those who consented to participation, 44 (14% of those eligible) completed the linkage.

Table 1 shows characteristics of OSCCAR cohort members who were successfully enrolled in and completed linkage with CCFA Partners compared with members who did not. The OSCCAR cohort is predominantly white (97%) and race did not appear to influence CCFA Partners enrollment and linkage. The mean age at diagnosis was 34 years in the linked group and 33 years in the unlinked groups. Similarly, disease type did not affect enrollment. Of those who completed linkage, 28 (63%) had CD and of those who did not 192 (60%) had CD. Fourteen (32%) and 122 (38%) of linked and unlinked subjects were diagnosed with UC, respectively.

OSCCAR participants who were successfully recruited into CCFA Partners were more educated (8 participants [17%] with a doctoral degree) than those who were not (7 participants [3%] with a doctoral degree, *P*=.01). Similarly, those who did not enroll in CCFA Partners were numerically more likely to have not attained a bachelor’s degree than those who did enroll; however, this was not statistically significant. There were no other significant demographic or clinical differences (eg, disease activity, disease behavior, disease location, medication use).

**Table 1:** Characteristics of OSCCAR cohort members who linked with CCFA Partners

			Linked (n=44)	Unlinked (n=224)	*P* value
Demographics					
	% Female		75	64.3	.22
	Race				1
		% White	97.7	96.6	
		% Black	2.3	3.4	
		% Hispanic or Latino	4.6	5.8	
		% Ashkenazi Jewish	11.9	4.2	.06
	Education				
		% Less than Bachelor’s degree	34.5	53.2	.07
		% Bachelor’s degree	41.4	31.4	.29
		% Master’s degree	6.9	12.2	.54
		% Doctoral degree	17.2	3.2	.01
	Occupation				.78
		% Paid occupation	54.6	50	
		% Disabled	2.3	4.9	
		% Other	43.2	45.1	
	Married		25	36.3	.17
	Ever smoked		18.2	17	.83
					
Disease course					
	Mean age at diagnosis (years)		34	33	.89
	Diagnosis				.39
		% Diagnosis of Crohn’s disease	63.4	59.8	
		% Diagnosis of ulcerative colitis	31.7	37.9	
		% Diagnosis of indeterminate colitis	4.9	2.3	
	% History of bowel resections		6.5	16.7	.18
	% Pyoderma gangrenosum		0	0	1
	% Erythema nodosum		0	0	1
	% Scleritis/episcleritis/iritis/uveitis		4.6	4	1
	% Sacroiliitis		4.6	1.3	.19
	% Family history of IBD		32	35	.73
					
Medications					
	% Steroid use^a^		4.9	3.7	.66
	% Aminosalicylate use^a^		41.5	37.5	.73
	% Immunomodulators^a^		12.2	19.4	.38
	% Biologic use^a^		24.4	25	1
					
Crohn’s disease					1
	% Ileal		20	21.5	
	% Colonic		28	27.7	
	% Ileo-colonic		52	50.1	
	% Concomitant upper disease		15.4	20.6	.79
% Fistulae			2.3	0.9	.42
Behavior					1
	% Inflammatory		88	83.2	
	% Stricturing		8	9.9	
	% Penetrating		4	6.9	
% Perianal disease			11.5	7.6	.85
Mean Harvey-Bradshaw Index^a^			2.3	2.5	.56
					
Ulcerative colitis					1
	% Proctitis		15.4	19.3	
	% Left-sided colitis		23.1	26.5	
	% Extensive colitis		61.5	54.2	
Mean Clinical Colitis Activity Index^a^			3.3	2.3	.17

^a^At the time of contact

### Share

**Figure 2:**
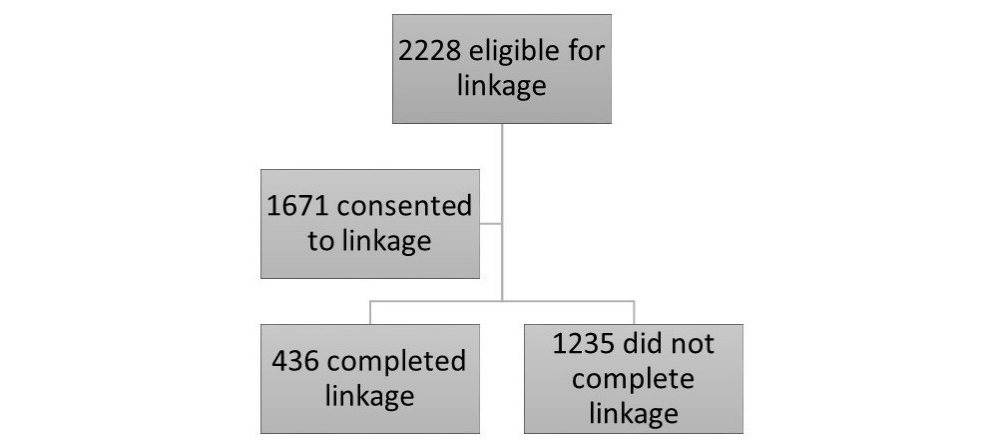
Recruitment efforts for the CCFA Partners/SHARE record linkage.

Of the SHARE participants from July 2012 to December 2014 who were approached to enroll in the CCFA Partners cohort, 1671 (75%) consented to linkage. However, only 24% (n=436) completed the linkage by enrolling in the Partners cohort (see Figure 2). Enrollment varied by study center. Table 2 shows characteristics of SHARE cohort members who were successfully enrolled in and completed linkage with CCFA Partners compared with members who did not. Gender was a notable difference: of those who linked, 263 (60%) were women, compared to 898 (50%) of those who did not link (*P*<.01). Linked subjects were predominantly white (96%; n=417); there were more blacks who did not link (6%; n=115; *P*<.01). Of those who linked, 49% (215) were using biological agents compared to 43% (767) of those who did not enroll (*P*=.02). There were no significant differences in patterns of use for other medication classes. Although there were no significant differences in disease location in CD, linked subjects had a mean HBI of 3.6 and those who did not link had a mean HBI of 4.4 (*P*<.01). Among those subjects with UC, 40% (176) of linked subjects had left-sided colitis compared to 27% (491) of those who did not link and 45% (196) of linked subjects had extensive colitis, compared to 60% (1066) of those who did not link (*P*<.01). However, there was no difference in the mean SCCAI score.

**Table 2:** Characteristics of SHARE cohort members who linked with CCFA Partners.

			Linked (n=436)	Unlinked (n=1792)	*P* value
Demographics					
	Mean age (years)		39	40	.10
	% Female		60.4	50.1	<.01
	Race				<.01
		% White	95.6	86.8	
		% Black	1.2	6.4	
		% Latino	2.8	3.5	.48
		% Ashkenazi Jewish	17.8	12.2	.15
	Ever smoked		29.3	34.2	.07
					
Disease course					
	Mean age at diagnosis (years)		29	29	.29
	Diagnosis				.20
		% Diagnosis of Crohn’s disease	65.4	60.8	
		% Diagnosis of ulcerative colitis	32.6	36.7	
		% Diagnosis of indeterminate colitis	2.1	2.6	
	% History of bowel resections		32.1	33.9	.48
	% Pyoderma gangrenosum		0.9	2.7	.03
	% Erythema nodosum		5.6	3.4	.03
	% Scleritis/episcleritis/iritis/uveitis		5.8	3.6	.04
	% Sacroiliitis		2.1	1.4	.28
	% Family history of IBD		24.5	22.6	.43
					
Medications					
	% Steroid use^a^		10.0	10.4	.83
	% Aminosalicylate use^a^		32.3	35.4	.23
	% Immunomodulators^a^		39.1	38.1	.70
	% Biological use^a^		49.3	42.8	.02
					
Crohn’s disease					.47
	% Ileal		29.3	25.7	
	% Colonic		20.4	20.8	
	% Ileo-colonic		50.4	53.5	
	% Concomitant upper disease		5.4	4.2	.37
Behavior					.11
	% Inflammatory		53.4	48.1	
	% Stricturing		27.8	27.2	
	% Penetrating		18.9	24.6	
% Perianal disease			14.6	14.6	.99
Mean Harvey-Bradshaw Index^a^			3.6	4.4	<.01
					
Ulcerative colitis					<.01
	% Proctitis		14.7	13.1	
	% Left-sided colitis		40.4	27.4	
	% Extensive colitis		44.9	59.5	
Mean Clinical Colitis Activity Index^a^			3.6	3.6	.52

^a^At the time of contact

## Discussion

### Principal Findings

This work represents a novel effort to link traditional cohorts of subjects with IBDs to an electronic cohort of IBD subjects. OSCCAR is the only US-based inception cohort of adult patients with IBD. SHARE is a large multicentered cohort of IBD subjects created to facilitate translational research. The accompanying biobank of specimens is an invaluable aspect of these cohorts. CCFA Partners is the largest US cohort of IBD subjects with a primary focus on patient-reported health data. The opportunity to leverage and enhance both cohorts through corecruitment and linkage of data assets is quite intuitive. The OSCCAR and SHARE cohorts are enriched through inclusion of an expanded array of patient-reported data. Similarly, CCFA Partners can provide a low-cost opportunity for the long-term follow-up of OSCCAR and SHARE participants in the event that funding for these cohorts ended. Conversely, for the portion of CCFA Partners participants who are coenrolled in OSCCAR or SHARE, the clinical data and biospecimens represent a valuable resource. Similar linkages between CCFA Partners and other clinical/translational cohorts will further expand the amount and types of data available to researchers.

As the findings reveal, it was a challenge to achieve linkage between the cohorts. Study staff worked diligently to use every contact as an opportunity to encourage subjects to participate in CCFA Partners. Although nearly 76% of the subjects who were eligible initially consented to enroll in CCFA Partners and link data, ultimately only 14% of those subjects who were eligible completed the linkage. The SHARE cohort had slightly more success with 24% of subjects linking to the CCFA Partners cohort. There are still a number of valuable lessons to be learned from this effort that both further inform the CCFA Partners study and may help inform future research endeavors involving record linkages.

CCFA Partners is an Internet-based cohort with most referrals coming from the CCFA rather than a population-based or randomly sampled cohort. The degree to which CCFA Partners is more broadly representative of the larger IBD population is largely unknown. However, systematic recruitment through the OSCCAR community-based cohort and SHARE multicentered cohort provided us the first opportunity to assess the generalizability of CCFA Partners. Education, race, gender, and disease activity for CD and location for UC appear to be factors impacting the decision to participate in CCFA Partners. This is important in understanding the generalizability of past and future findings learned through CCFA Partners. We also note that OSCCAR or SHARE participants who did not have access to the Internet were excluded from this linkage study. Therefore, additional demographic or clinical differences between the CCFA Partners cohort and the general IBD population may exist and will need to be evaluated in subsequent studies.

Data exploring factors contributing to participation in cohort studies of chronic diseases are scarce. Our findings indicate that education level was a very important driver of cohort linkage. In the OSCCAR study, 17% of participants who successfully linked possessed a doctoral degree compared with 3% of participants who did not complete the linkage. Furthermore, a higher percentage of participants who did not link had less than a bachelor’s degree. One possible explanation for this difference is that those with a doctoral degree valued the importance of research endeavors and made it a priority to complete the linkage. Education level was not available for the SHARE cohort.

The SHARE experience revealed that gender and race may play a role in completing linkage to an Internet-based cohort, with a significantly higher percentage of women and white people completing the linkage. It is possible that those who linked have less aggressive disease than those who did not link. Among CD patients, although there were no differences in disease phenotypes, those who linked had a lower mean HBI. Among UC patients, those who linked had less-extensive disease, even though the mean SCCAI was the same between both groups.

To date, there is only one study examining factors in participation in cohort studies of IBDs. A Swiss IBD cohort reported that factors associated with nonresponse to an initial survey were younger age, male gender, and depression in CD only and longer disease duration in UC only. [31] These differences were not noted in our experience. HIV/AIDS is the disease process in which study participation has been most studied. Unemployment, female gender, higher education, and higher baseline CD4 counts predicted likelihood of participation in HIV cohort studies [22-34]. A study of childhood cancer survivors concluded that there were no substantive differences explaining nonparticipation in a lifetime cohort study [35], similar to our findings. Other groups described factors that contribute to participation in studies in general. A cohort study of employees at a large French utility company demonstrated that male sex, being married, having children, being a manager, and geography were predictors of participation [36]. However, this study likely cannot be generalized to cohort studies of disease processes.

We speculate that recruiting participants into CCFA Partners at the time of their enrollment in OSCCAR (the time of their initial diagnosis) would have likely yielded more success in linkage. The OSCCAR cohort started enrollment 3 years before the initiation of the Partners cohort. It is possible that research fatigue was a factor explaining the poor linkage. It is also possible that the farther participants get from their diagnosis, the more their initial enthusiasm to participate in research wanes. SHARE participants were recruited at the time of the initial SHARE consent. Although similar proportions of subjects consented to enrollment, the SHARE experience demonstrated slightly more success with completing the linkage. Future investigators establishing new cohorts may want to anticipate potential linkages and incorporate them into the initial procedures at the beginning of the cohort. Indeed this is already being implemented with CCFA Partners recruitment and linkage incorporated into the design of the recently funded IBD Plexus Prospective Clinical Cohort.

The main strength of this study was the leveraging of the existing infrastructure of 3 well-developed cohorts. The primary limitation, aside from the disappointing recruitment and linkage results, is that we were not able to further explore reasons for noncompletion of CCFA Partners enrollment and linkage. Although interviews and surveys of OSCCAR and SHARE participants to inquire about reasons for nonparticipation may have been informative, we decided not to pursue this due to a concern that this might jeopardize continued participation in the cohorts themselves.

### Conclusions

The linking of complementary cohorts may be a cost-effective strategy for expanding research infrastructure and enabling studies that might not be possible with either cohort alone. The regulatory, legal, and technical challenges in creating and implementing such linkages are not insurmountable; however, in this pilot study, we observed that recruitment itself was the substantial obstacle. Therefore, recruitment efforts for cohort linkages need to be balanced against the potential returns. Based on our experience, we would recommend a simultaneous, rather than sequential, strategy for corecruitment for subsequent attempts at cohort linkage. We believe that the lessons learned from this novel effort serve well to inform future linkages both in IBD and other chronic diseases.
